# Supplementary material for: Novel Driver Strength Index highlights important cancer genes in TCGA PanCanAtlas patients
Source: PeerJ. 2022 Aug 11;10:e13860. doi: 10.7717/peerj.13860 (PMC9375969; doi:10.7717/peerj.13860)
Supplement: Supplemental Information 2 [file peerj-10-13860-s002.zip › COHORTS/genes plots/stage/2021_8_16_14_9_distribution_stage_females_IV.pdf]

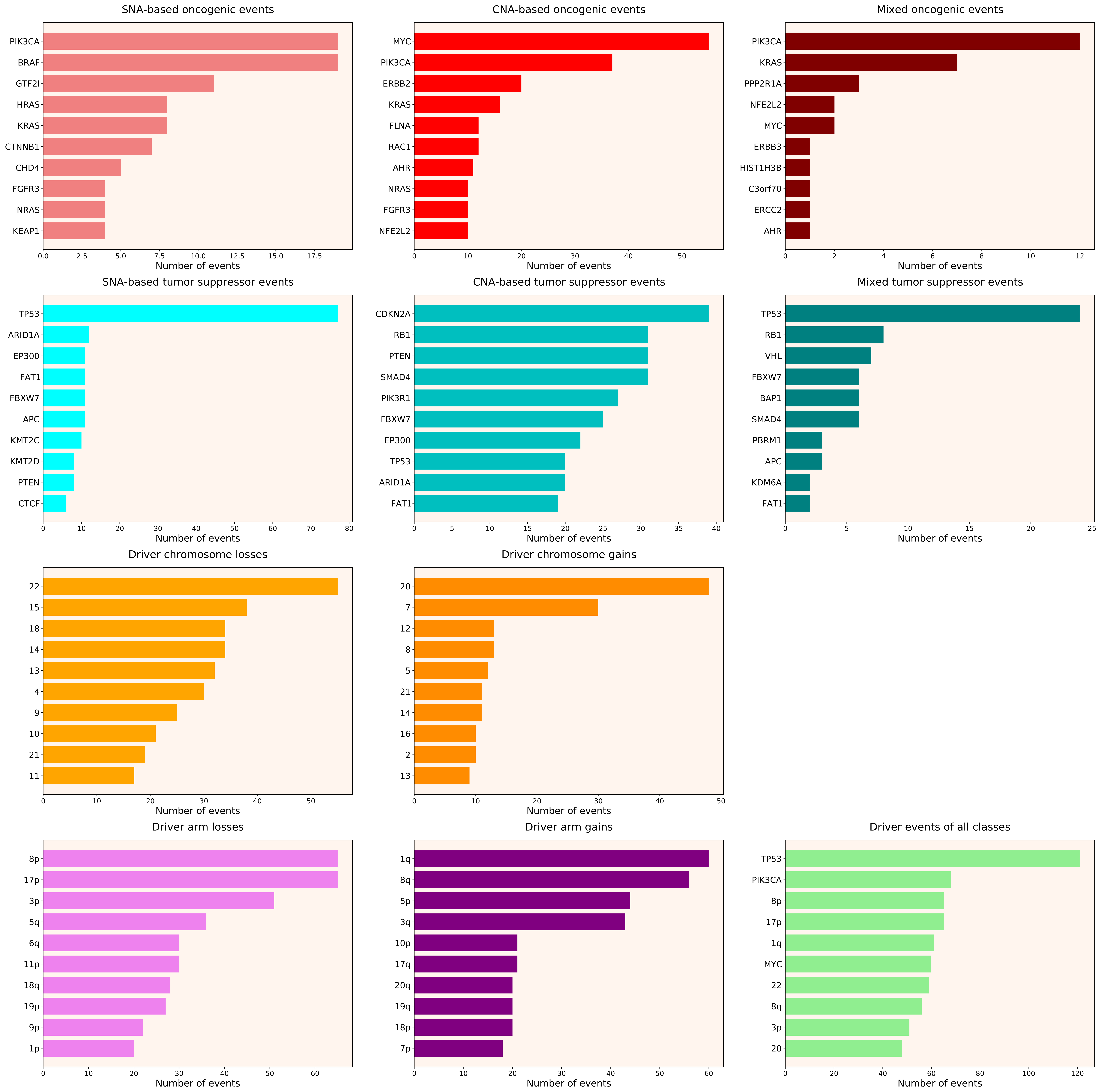

SNA-based oncogenic events
PIK3CA
BRAF
GTF2I
HRAS
KRAS
CTNNB1
CHD4
FGFR3
NRAS
KEAP1
0.0 2.5 5.0 7.5 10.0 12.5 15.0 17.5
Number of events
CNA-based oncogenic events
MYC
PIK3CA
ERBB2
KRAS
FLNA
RAC1
AHR
NRAS
FGFR3
NFE2L2
0 10 20 30 40 50
Number of events
Mixed oncogenic events
PIK3CA
KRAS
PPP2R1A
NFE2L2
MYC
ERBB3
HIST1H3B
C3orf70
ERCC2
AHR
0 2 4 6 8 10 12
Number of events
SNA-based tumor suppressor events
TP53
ARID1A
EP300
FAT1
FBXW7
APC
KMT2C
KMT2D
PTEN
CTCF
0 10 20 30 40 50 60 70 80
Number of events
CNA-based tumor suppressor events
CDKN2A
RB1
PTEN
SMAD4
PIK3R1
FBXW7
EP300
TP53
ARID1A
FAT1
0 5 10 15 20 25 30 35 40
Number of events
Mixed tumor suppressor events
TP53
RB1
VHL
FBXW7
BAP1
SMAD4
PBRM1
APC
KDM6A
FAT1
0 5 10 15 20 25
Number of events
Driver chromosome losses
22
15
18
14
13
4
9
10
21
11
0 10 20 30 40 50
Number of events
Driver chromosome gains
20
7
12
8
5
21
14
16
2
13
0 10 20 30 40 50
Number of events
Driver arm losses
8p
17p
3p
5q
6q
11p
18q
19p
9p
1p
0 10 20 30 40 50 60
Number of events
Driver arm gains
1q
8q
5p
3q
10p
17q
20q
19q
18p
7p
0 10 20 30 40 50 60
Number of events
Driver events of all classes
TP53
PIK3CA
8p
17p
1q
MYC
22
8q
3p
20
0 20 40 60 80 100 120
Number of events
